# Additive Effects of Obesity on Myocardial Microcirculation and Left Ventricular Deformation in Essential Hypertension: A Contrast-Enhanced Cardiac Magnetic Resonance Imaging Study

**DOI:** 10.3389/fcvm.2022.831231

**Published:** 2022-03-24

**Authors:** Pei-Lun Han, Xue-Ming Li, Li Jiang, Wei-Feng Yan, Ying-Kun Guo, Yuan Li, Kang Li, Zhi-Gang Yang

**Affiliations:** ^1^West China Biomedical Big Data Center, West China Hospital, Sichuan University, Chengdu, China; ^2^Department of Radiology, West China Hospital, Sichuan University, Chengdu, China; ^3^Department of Radiology, West China Second University Hospital, Sichuan University, Chengdu, China

**Keywords:** hypertension, obesity, left ventricular deformation, myocardial perfusion, magnetic resonance imaging

## Abstract

**Objective:**

The combination of hypertension and obesity is a major cause of cardiovascular risk, and microvascular changes and subclinical dysfunction should be considered to illustrate the underlying mechanisms and early identification, thereby developing targeted therapies. This study aims to explore the effect of obesity on myocardial microcirculation and left ventricular (LV) deformation in hypertensive patients by cardiac magnetic resonance (CMR).

**Methods:**

This study comprised 101 hypertensive patients, including 54 subjects with a body mass index (BMI) of 18.5–24.9 kg/m^2^ and 47 subjects with a BMI ≥25 kg/m^2^, as well as 55 age- and sex-matched controls with a BMI of 18.5–24.9 kg/m^2^. Myocardial perfusion indicators [upslope, time to maximum signal intensity (TTM), maximum signal intensity (Max SI)] and LV strains [radial, circumferential, and longitudinal global peak strain (PS), peak systolic strain rate (PSSR), and peak diastolic strain rate (PDSR)] were measured.

**Results:**

Upslope was numerically increased in obese patients but statistically decreased in non-obese patients compared with controls. Longitudinal PS deteriorated significantly and gradually from controls to non-obese and obese hypertensive patients. Longitudinal PSSR and PDSR were significantly decreased in obese hypertensive patients compared with the other two groups. BMI was associated with upslope (β = −0.136, *P* < 0.001), Max SI (β = −0.922, *P* < 0.001), longitudinal PSSR (β = 0.018, *P* < 0.001), and PDSR (β = −0.024, *P* = 0.001). Myocardial perfusion was independently associated with longitudinal PSSR (TTM: β = 0.003, *P* = 0.017) and longitudinal PDSR (upslope: β = 0.067, *P* = 0.020) in hypertension.

**Conclusion:**

Obesity had adverse effects on microvascular changes and subclinical LV dysfunction in hypertension, and BMI was independently associated with both myocardial perfusion and LV deformation. Impaired myocardial perfusion was independently associated with subclinical LV dysfunction in hypertension.

## Introduction

Hypertension and obesity are well-recognized global health concerns, and the two conditions often coexist (1–3). Both hypertension and obesity are associated with an increased risk of cardiovascular morbidity and mortality in isolation, and when combined the cardiovascular risk increases (4). Myocardial microcirculatory damage is an important mechanism of myocardial impairment (5) that is associated with cardiac dysfunction (6), poor prognosis (7), and adverse outcomes (8). Although abnormalities of myocardial perfusion and left ventricular (LV) function have been demonstrated separately in hypertension (9, 10) and obesity (11, 12) separately, their combined effects are still poorly investigated. Elucidation of the interaction between obesity and hypertension may provide insights into mechanisms underlying the impairment of myocardial microcirculation and cardiac function, and may help in identifying patient subgroups that could benefit from early prevention and treatment.

Cardiac magnetic resonance (CMR) imaging has been increasingly used to evaluate myocardial microcirculation and cardiac function with high reproducibility. First-pass CMR imaging allows non-invasive assessment of myocardial perfusion during the transit of contrast agent and has been increasingly used in recent years to detect microvascular dysfunction (13, 14). In addition, conventional functional parameters are not sensitive enough to detect minor abnormalities of LV function, such as left ventricular ejection fraction (LVEF), which may mask the impairment in the preclinical stage (15). CMR feature tracking (CMR-FT) can drive LV deformation indicators using only clinical routine cine images, and has been well established as a sensitive technique for evaluating subclinical LV dysfunction (16, 17).

Therefore, we aimed to evaluate myocardial microcirculation and LV function by CMR first-pass perfusion and CMR-FT imaging in hypertensive patients without or with obesity, as well as to explore the relationship between myocardial perfusion, LV strains and BMI.

## Materials and Methods

### Ethical Considerations

This study was approved by the Biomedical Research Ethics Committee of the West China hospital of Sichuan University (Approval No. 2019-756) and was conducted in accordance with institutional guidelines. All patients gave their informed consent for contrast-enhanced CMR examination. All patient information was used only for research purposes.

### Study Population

We retrospectively identified patients with essential hypertension from consecutive hospitalized subjects who underwent contrast-enhanced CMR for suspected cardiomyopathy at our hospital from January 2016 to May 2021. Hypertension was defined as clinical systolic blood pressure (SBP) ≥140 mmHg and/or diastolic blood pressure (DBP) ≥90 mmHg or a previous diagnosis of essential hypertension or usage of antihypertensive medication. The exclusion criteria included coronary artery disease, myocardial infarction, severe arrhythmias, symptoms of heart failure or LVEF <50%, cardiomyopathy, severe valvular disease, congenital heart disease, diabetes diseases, estimated glomerular filtration rate (e-GFR) <30 ml/min, severe hepatopulmonary dysfunction, underweight (BMI <18.5 kg/m^2^), and poor image quality. Ultimately, 101 hypertensive patients (51 [50.49%] females; mean age, 54.56 ± 15.33 years) were included in the final analysis. Then, the hypertensive patients were categorized into two groups (without and with obesity) according to World Health Organization (WHO) BMI standards (18) for Asian populations. The hypertensive patients without obesity group comprised those with a BMI of 18.5 kg/m^2^–24.9 kg/m^2^ (*n* = 54), and the hypertensive patients with obesity group comprised those with a BMI ≥25.0 kg/m^2^ (*n* = 47). For comparison, 55 age- and sex- matched controls (33 [60.0%] females; mean age, 53.54 ± 10.08 years) were identified though subjects who underwent contrast-enhanced CMR during the study period. All these individuals had BMIs of 18.5 kg/m^2^–24.9 kg/m^2^ and no evidence of hypertension. Moreover, the aforementioned exclusion criteria for the hypertensive group also applied to the control group. Demographic and clinical data within 1 month of the CMR examination were collected through a review of electronic medical charts.

### Cardiac Magnetic Resonance Imaging Acquisition

Cardiac magnetic resonance scans were performed on a 3.0 T whole-body scanner MAGNETOM Trio Tim (Siemens Medical Solutions, Erlangen, Germany). Patients were placed in the supine position with a dedicated two-element cardiac-phased array coil attached.

A standard ECG-triggering device was simultaneously used. Localizers were used to determine the cardiac axes. To achieve complete and high-quality LV coverage, CMR images were acquired from the base to the apex during multiple breath-holding periods. To analyze cardiac function and myocardial strain, a balanced steady-state free-precession (bSSFP) sequence (repetition time [TR]/echo time [TE]: 2.81/1.22 ms, flip angle: 40°, slice thickness: 8 mm, field of view [FOV]: 250 × 300 mm, matrix size: 208 × 139) was used to obtain 8–12 continuous cine images of the long-axis, short-axis, 2-chamber, and 4-chamber views. For perfusion imaging, a contrast dose of 0.2 ml/kg gadobenate dimeglumine (MultiHance 0.5 mmol/mL; Bracco, Milan, Italy) was injected into the right antecubital vein using an automated injector (Stellant, MEDRAD, Indianola, PA, United States) at a flow rate of 2.5–3.0 ml/s, followed by a 20 ml saline flush at a rate of 3.0 ml/s. Resting perfusion images were acquired concurrently with intravenous contrast agents in three stand- and short-axis slices (apical, middle, and basal) and in one 4-chamber view slice by inversion-recovery echo-planar sequence (TR/TE: 163/1.12 ms, flip angle: 10°, slice thickness: 8 mm, FOV: 360 × 270 mm, matrix size: 256 × 192). Each set of first-pass perfusion images was acquired in 80 cardiac cycles. To exclude myocardial infarction, late gadolinium enhancement (LGE) imaging was obtained at an average of 10–15 min after contrast injection by segmented-turbo-FLASH–phase-sensitive inversion recovery (PSIR) sequence (TR/TE: 750/1.18 ms, flip angle: 40°, slice thickness: 8 mm, FOV: 400 × 270 mm, matrix size: 256 × 148).

### Cardiac Magnetic Resonance Imaging Analysis

All CMR data were transferred to a dedicated software program (CVI42 version 5.9.1, Circle Cardiovascular Imaging, Inc., Calgary, Canada) and measured by two experienced (at least 2 years of CMR experience) investigators blinded to the clinical profiles of the subjects. For measurement of left ventricular (LV) function parameters, we manually delineated the endocardial and epicardial contours in serial short-axis slices at the end-diastolic and end-systolic, and then left ventricular end diastolic volume (LVEDV), left ventricular end systolic volume (LVESV), left ventricular stroke volume (LVSV), LVEF and left ventricular mass (LVM) were calculated automatically. LVEDV, LVESV and LVM were indexed to body surface area (LVEDVI, LVESVI and LVMI, respectively). The LV remodeling index was calculated as LVM divided by LVEDV.

For semiquantitative analysis of LV myocardial perfusion, we manually delineated the endocardial contours, epicardial contours, and a region of interest drawn in the LV blood pool in all of the first-pass perfusion images of the basal, mid and apical short-axis slices. A 16-segment mode (Bull’s eye plot) was constructed based on AHA standard segmentation recommendations, including six basal segments, six middle segments, and four apical segments (19). Subsequently, a myocardial signal intensity-time curve was generated, and the LV segmental perfusion parameters [upslope, max signal intensity (Max SI), and time to maximum signal intensity (TTM)] were obtained automatically.

Cardiac magnetic resonance feature tracking (CMR-FT) was used for the analysis of LV myocardial strain. We manually delineated the endocardial and epicardial contours in long-axis 2-chamber, 4-chamber, and serial short-axis slices at the end-diastole phase, which was the reference phase, in a 3-dimensional (3D) tissue tracking module. Then, the LV global myocardial strain parameters (radial, circumferential, and longitudinal global peak strain (PS), peak systolic strain rate (PSSR), and peak diastolic strain rate (PDSR)) were acquired automatically.

### Reproducibility of Left Ventricular Strain and First-Pass Myocardial Perfusion Parameters

After 1 month, 30 patients (20 hypertensive subjects, 10 controls) were randomly selected and LV strain and first-pass myocardial perfusion parameters were measured again by the same radiologist to evaluate the intraobserver variability. The parameters were measured again as above by a second blinded investigator to determine the interobserver variability.

### Statistical Analysis

All statistical analyses were performed with R version 3.6.3 (The R Foundation, Vienna, Austria). Continuous variables are presented as the mean ± standard deviation and categorical variables are presented as frequencies (%). The Kolmogorov–Smirnov test was used to assess the normality of the distribution of continuous variables. Normally distributed continuous variables among groups were compared by one-way analysis of variance (ANOVA) followed by the least-significant difference (LSD) test. Comparisons of non-normally distributed continuous variables among groups were performed by the Kruskal-Wallis rank test. Categorical variables were compared by the Chi-square test. Correlations between myocardial perfusion and LV strain parameters were analyzed by Pearson correlation analysis. Univariable linear regression analyses were performed to demonstrate the relationship between candidate factors and myocardial perfusion/LV strain parameters. Age, sex and variables with a *P*-value <0.1 in the univariable analyses were entered into a stepwise multivariable linear regression analysis. The inter- and intraobserver variabilities for reproducibility were evaluated using the intraclass correlation coefficient (ICC). For all statistical analyses, a *P*-value <0.05 was considered statistically significant.

## Results

### Baseline Characteristics

Table 1 presents the demographic and clinical characteristics of the study population. BMI and BSA were significantly higher in hypertensive patients with obesity than in the other two groups (all *P* < 0.05). Office SBP and DBP were significantly higher in hypertensive patients with or without obesity than in controls (all *P* < 0.05); office DBP was significantly higher in hypertensive patients with obesity than in those without obesity (*P* = 0.003). High-density lipoprotein (HDL) was significantly increased in hypertensive patients without obesity compared with the other two groups. Calcium channel blocker use was more common in obese patients than in non-obese patients (*P* = 0.014). No significant differences were found in in sex, age, heart rate or other laboratory data among the three groups. There were no significant differences in other medication usage or duration of hypertension between hypertensive patients with and without obesity.

**TABLE 1:** Baseline characteristics.

	Controls (*n* = 55)	Hypertensive patients without obesity (*n* = 54)	Hypertensive patients with obesity (*n* = 47)	P
**Demographics**				
Age, years	53.54 ± 10.08	54.15 ± 16.18	55.02 ± 14.46	0.862
Sex				0.288
Male, *n* (%)	22 (40.0%)	24 (44.4%)	26 (55.3%)	
Female, *n* (%)	33 (60.0%)	30 (55.6%)	21 (44.7%)	
BMI, kg/m^2^	22.49 ± 1.75	22.70 ± 1.46	27.57 ± 2.20*§	<0.001
BSA, m^2^	1.62 ± 0.13	1.59 ± 0.14	1.79 ± 0.18*§	<0.001
**Hemodynamic variables**				
Heart rate, bpm	71.03 ± 10.52	75.98 ± 16.46	77.02 ± 15.14	0.161
Office SBP, mmHg	114.55 ± 10.33	138.98 ± 23.57*	143.13 ± 17.78*	<0.001
Office DBP, mmHg	73.49 ± 10.98	82.54 ± 16.59*	91.34 ± 15.68*§	<0.001
Laboratory data				
TG, mmol/L	1.46 ± 0.81	1.61 ± 1.48	1.98 ± 1.77	0.156
TC, mmol/L	4.53 ± 1.08	4.36 ± 0.82	4.66 ± 1.08	0.301
HDL, mmol/L	1.37 ± 0.38	1.61 ± 1.42	1.25 ± 0.45*§	0.018
LDL, mmol/L	2.68 ± 1.00	2.41 ± 0.68	2.78 ± 0.86	0.082
eGFR, mL/min/1.73 m^2^	90.62 ± 13.95	91.16 ± 24.70	91.98 ± 20.14	0.965
**Medication usage**				
ACEI, *n* (%)	–	4 (7.4%)	4 (8.5%)	1.000
ARB, *n* (%)	–	17 (31.5%)	14 (29.8%)	0.854
Beta-blockers, n (%)	–	18 (33.3%)	15 (31.9%)	0.880
CCB, *n* (%)	–	19 (35.2%)	28 (59.6%)§	0.014
Diuretics, n (%)	–	4 (7.4%)	4 (8.5%)	1.000
Duration of hypertension, years	–	5.88 ± 7.93	6.23 ± 5.35	0.124

*BMI, body mass index; BSA, body surface area; SBP, systolic blood pressure; DBP, diastolic blood pressure; TG, plasma triglycerides; TC, total cholesterol; HDL, high-density lipoprotein; LDL, low-density lipoprotein; eGFR, estimated glomerular filtration rate; ACEI, angiotensin-converting enzyme inhibitors; ARB, angiotensin receptor blocker; CCB, Calcium channel blocker*

*P < 0.05 versus controls.

*§P < 0.05 versus hypertensive patients without obesity.*

### Comparison of Cardiac Magnetic Resonance Findings Between Groups

As shown in Table 2, LVEDVI and LVESVI were significantly higher in hypertensive patients without obesity than in the other two groups (all *P* < 0.05). LVMI was significantly higher in hypertensive patients than in controls (both *P* < 0.05). The LV remodeling index was gradually increased in non-obese and obese hypertensive patients compared with controls (all *P* < 0.005).

**TABLE 2:** Comparisons of CMR findings between controls, non-obese and obese patients.

	Controls (*n* = 55)	Hypertensive patients without obesity (*n* = 54)	Hypertensive patients with obesity (*n* = 47)	P
**Conventional LV function**				
LVEF,%	64.45 ± 5.08	62.57 ± 6.06	62.65 ± 7.10	0.239
LVEDVI, ml/m^2^	74.51 ± 13.58	84.24 ± 19.66*	74.00 ± 19.47^§^	0.004
LVESVI, ml/m^2^	26.83 ± 6.27	34.19 ± 11.06*	28.32 ± 10.03^§^	<0.001
LVSV, ml	77.53 ± 15.96	81.56 ± 21.85	82.88 ± 24.26	0.757
LVMI, g/m^2^	47.02 ± 14.26	61.00 ± 19.35*	62.06 ± 23.26*	<0.001
LVremodeling index, g/mL	0.64 ± 0.18	0.73 ± 0.19*	0.85 ± 0.25^*§^	<0.001
**Myocardial perfusion**				
Upslope	2.56 ± 0.86	2.83 ± 0.89	1.90 ± 0.60^*§^	<0.001
TTM, s	26.94 ± 10.04	26.94 ± 12.53	32.05 ± 13.65	0.059
Max SI	23.26 ± 6.32	26.24 ± 7.96	20.61 ± 6.33^§^	0.001
**LV strain**				
*PS,%*				
Radial	35.81 ± 8.31	34.80 ± 9.14	33.17 ± 8.83	0.221
Circumferential	−20.58 ± 2.66	−20.20 ± 3.25	−20.31 ± 3.14	0.798
Longitudinal	−14.32 ± 2.28	−13.10 ± 3.12*	−11.72 ± 2.82^*§^	<0.001
*PSSR, 1/s*				
Radial	1.92 ± 0.52	1.92 ± 0.63	1.81 ± 0.71	0.619
Circumferential	−1.00 ± 0.36	−1.04 ± 0.26	−1.06 ± 0.23	0.813
Longitudinal	−0.84 ± 0.20	−0.78 ± 0.22	−0.68 ± 0.17^*§^	<0.001
*PDSR, 1/s*				
Radial	−2.40 ± 0.89	−2.17 ± 0.88	−2.00 ± 0.61	0.086
Circumferential	1.23 ± 0.25	1.15 ± 0.30	1.07 ± 0.26*	0.018
Longitudinal	0.91 ± 0.24	0.82 ± 0.22	0.67 ± 0.22^*§^	<0.001

LV, left ventricular; EF, ejection fraction; EDVI, end diastolic volume index; ESVI, end systolic volume index; SV, stroke volume; MI, mass index; TTM, time to maximum signal intensity; Max SI, maximum signal intensity; PS, peak strain; PSSR, peak systolic strain rate; PDSR, peak diastolic strain rate *P < 0.05 versus controls ^§^P < 0.05 versus hypertensive patients without obesity.

**FIGURE 1:**
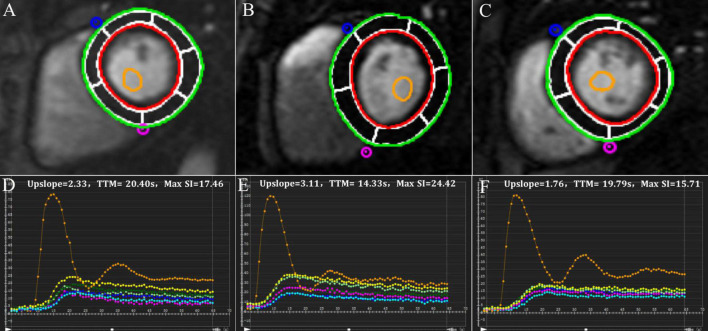
Representative first-pass myocardial perfusion MR images and signal intensity-time curves of a control subject **(A,D)**, a hypertensive patient without obesity **(B,E)** and a hypertensive patient with obesity **(C,F)**.

**FIGURE 2:**
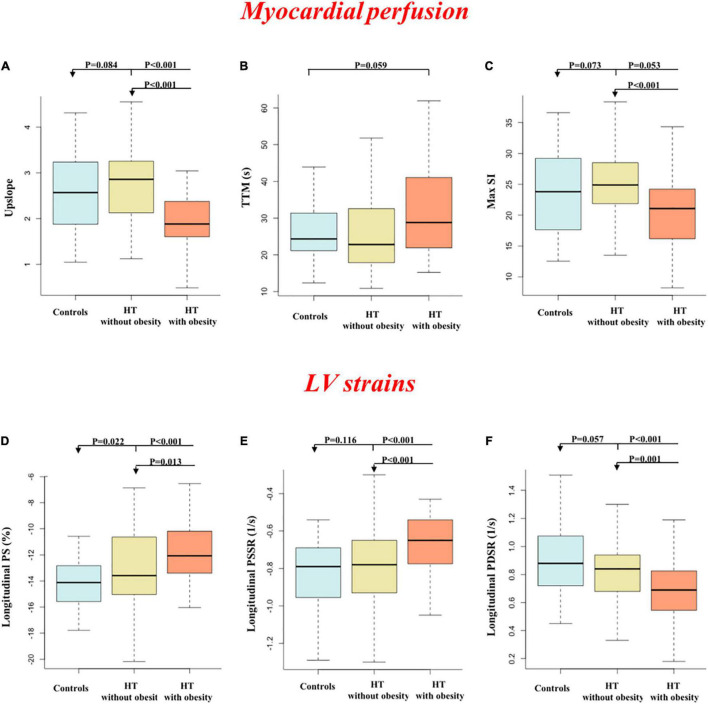
Comparisons of first-pass perfusion **(A–C)** and left ventricular strains **(D–F)** between groups.

Hypertensive patients without obesity had numerically higher upslope and Max SI values than controls (upslope: 2.83 ± 0.89 vs. 2.56 ± 0.86, *P* = 0.084; Max SI: 26.24 ± 7.96 vs. 23.26 ± 6.32, *P* = 0.073). Hypertensive patients with obesity had a significantly reduced upslope compared with the other two groups, and a significantly decreased Max SI compared with patients without obesity (all *P* < 0.05) (Figures 1, 2).

Longitudinal PS significantly and gradually deteriorated from controls to patients without obesity to patients with obesity (all *P* < 0.05). Longitudinal PSSR and PDSR were significantly decreased in hypertensive patients with obesity compared with the other two groups (all *P* < 0.05). In addition, circumferential PDSR was significantly decreased in hypertensive patients with obesity compared with controls (*P* = 0.005). No significant differences were seen in other parameters among the three groups.

### Associations Between Perfusion and Deformation Parameters in Non-obese and Obese Hypertensive Patients

Among hypertensive patients, TTM was correlated with all strain parameters (all *P* < 0.05) (Supplementary Figure 1). In addition, there were significant correlations between upslope and longitudinal PS, PSSR and PDSR (all *P* < 0.05). Univariable linear regression analyses between potential influencing factors and perfusion/strain parameters are shown in Supplementary Table 1.

**FIGURE 3:**
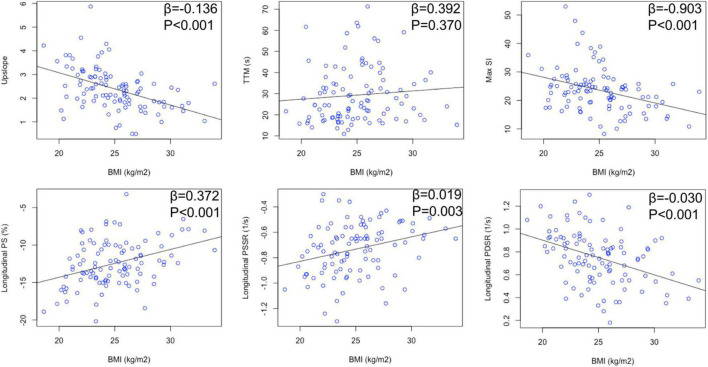
Relationship between BMI and myocardial perfusion/LV deformation in hypertensive patients.

**FIGURE 4:**
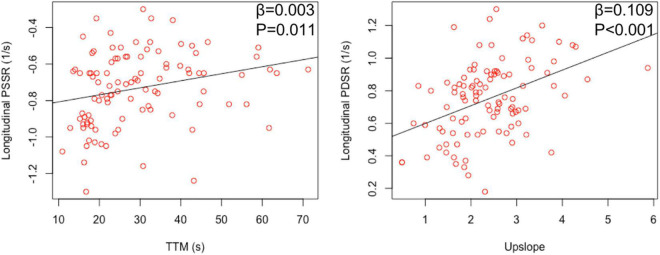
Relationship between myocardial perfusion and LV deformation in hypertensive patients.

looseness1 Multivariable linear regression analyses (Table 3) showed that BMI was significantly associated with myocardial perfusion (upslope: β = −0.136, *P* < 0.001, model R^2^ = 0.214; Max SI: β = −0.922, *P* < 0.001, model *R*^2^ = 0.172) and LV strains (longitudinal PSSR: β = 0.018, *P* < 0.001, model R^2^ = 0.245; longitudinal PDSR: β = −0.024, *P* = 0.001, model *R*^2^ = 0.278) in hypertensive patients (Figure 3). After adjustment for perfusion, there was a significant association between BMI and longitudinal PS after adjustment for perfusion (β = 0.493, *P* = 0.022, model R^2^ = 0.496). The association between BMI and longitudinal PSSR remained significant (β = 0.016, *P* = 0.009, model *R*^2^ = 0.326), while the association between BMI and PDSR was absent (β = −0.013, *P* = 0.132, model R^2^ = 0.385). In addition, multivariable linear regression analyses including both BMI and all perfusion parameters revealed that myocardial perfusion was significantly associated with longitudinal PSSR (TTM: β = 0.003, *P* = 0.017) and longitudinal PSDR (upslope: β = 0.067, *P* = 0.020) in hypertension (Figure 4).

**TABLE 3:** Multivariable linear regression analyses in hypertensive patients.

	Upslope			TTM			Max SI		
Model 1	β (95% CI)	P	R^2^	β	P	R^2^	β	P	R^2^
BMI	−0.136 (−0.189, −0.084)	<0.001*	0.214	–	–	0.067	−0.922 (−1.385, −0.458)	<0.001*	0.172

	**Longitudinal PS**			**Longitudinal PSSR**			**Longitudinal PDSR**		
	**β (95% CI)**	***P***	***R* ^2^**	β	***P***	***R* ^2^**	β	***P***	***R* ^2^**

**Model 2**									
BMI	–	–	0.385	0.018 (0.005–0.030)	<0.001*	0.245	−0.024 (−0.037, −0.010)	0.001*	0.278
**Model 3**									
BMI	0.493 (0.079, 0.906)	0.022*	0.496	0.016 (0.004, 0.028)	0.009*	0.326	−0.013 (−0.028, 0.002)	0.132	0.385
Upslope	–	–		–	–		0.067 (0.011, 0.124)	0.020*	
TTM	–	–		0.003 (0.000, 0.006)	0.017*		−0.002 (−0.006, 0.001)	0.156	
Max SI	0.184 (−0.035, 0.402)	0.095		–	–		–	–	

*BMI, body mass index; PDSR, peak diastolic strain rate; PSSR, peak systolic strain rate; PS, peak strain; TTM, time to maximum signal intensity; Max SI, maximum signal intensity.*

*Age, sex, and factors with p < 0.1 in the univariable analysis were included in the multivariable analysis.*

*Model 1: Association between BMI and perfusion parameters in hypertension.*

*Model 2: Association between BMI and longitudinal strain/strain rates in hypertension.*

*Model 3: Association between perfusion parameters and longitudinal strain/strain rates in hypertension.*

*P < 0.05.

*–Factors not incorporated into the final regression equation.*

### Intra- and Interobserver Variability

There was excellent intra- and interobserver variability for first-pass perfusion (ICC = 0.900–0.980) and LV deformation indicators (ICC = 0.850–0.977) (Table 4).

**TABLE 4:** Inter- and intra-observer variability of CMR first-pass perfusion and LV strain parameters.

Variable	Intra-observer (*n* = 30)		Inter-observer (n = 30)	
	ICC	95%CI	ICC	95%CI
**First-pass perfusion parameters**				
Upslope	0.960	0.917–0.981	0.980	0.959–0.991
TTM	0.951	0.899–0.976	0.953	0.903–0.977
Max SI	0.900	0.801–0.951	0.973	0.944–0.987
**Strain parameters**				
*PS,%*				
Radial	0.950	0.899–0.976	0.930	0.858–0.966
Circumferential	0.924	0.847–0.917	0.854	0.716–0.928
Longitudinal	0.946	0.942–0.987	0.857	0.721–0.929
***PSSR, 1/s***				
Radial	0.887	0.777–0.945	0.879	0.762–0.941
Circumferential	0.938	0.874–0.970	0.842	0.694–0.921
Longitudinal	0.973	0.944–0.987	0.922	0.842–0.962
*PDSR, 1/s*				
Radial	0.977	0.952–0.989	0.850	0.709–0.926
Circumferential	0.889	0.780–0.945	0.877	0.757–0.939
Longitudinal	0.932	0.863–0.967	0.925	0.849–0.964

*LV, left ventricular; EF, ejection fraction; EDVI, end diastolic volume index; ESVI, end systolic volume index; SV, stroke volume; MI, mass index; TTM, time to maximum signal intensity; Max SI, maximum signal intensity; PS, peak strain; PSSR, peak systolic strain rate; PDSR, peak diastolic strain rate.*

## Discussion

In this work, we used contrast-enhanced CMR and found that (1) myocardial perfusion was slightly increased in hypertensive patients without obesity but significantly decreased in hypertensive patients with obesity; (2) subclinical LV function gradually decreased from controls to hypertensive patients without and with coexisting obesity; and (3) myocardial microcirculatory damage was independently associated with subclinical LV dysfunction in hypertensive patients.

### The Combined Effects of Obesity and Hypertension on Myocardial Microcirculatory Damage

The pathophysiology of cardiomyopathy related to obesity and hypertension is complex and multifactorial. Among these, microvascular abnormalities are an important disorder in both conditions with certain common pathological changes: endothelial dysfunction, microvascular remodeling and rarefaction (20). Some previous studies revealed no obvious difference in the resting myocardial perfusion between hypertensive and normotensive subjects (21, 22), while other studies reported significantly increased resting myocardial perfusion in hypertensive patients due to an adaptive mechanism (23, 24). Our results showed slightly increased resting myocardial perfusion in hypertensive patients without obesity compared with controls, but this difference did not reach statistical significance. Regarding the effects of obesity alone on myocardial microvascular damage, Bajaj et al. (11) reported that coronary microvascular dysfunction was independently associated with elevated BMI in patients with suspected coronary artery disease based on clinical symptoms. Such association was also confirmed in persons without traditional coronary artery disease risk factors (25). Considering the shared pathophysiology of obesity and hypertension above, we hypothesized that the obesity status contributes to the aggravation of the microcirculatory dysfunction by hypertension.

Although a combined effect of obesity and diabetes on damage to myocardial microcirculation has been reported (14), whether such an effect would be observed among obese individuals with hypertension is still unclear. Our findings demonstrated that hypertensive patients with obesity had significantly decreased perfusion compared with both controls and hypertensive patients without obesity even in the resting state, suggesting that the coexistence of obesity may amplify the microcirculatory damage in hypertension. This result may help to provide a better understanding of the possible mechanisms of the combined effect of hypertension and obesity on the myocardium. In addition, coronary microvascular dysfunction was reported to be a better risk marker of adverse outcomes than BMI and other traditional risk factors (11), which provides a direction for tailoring therapeutic strategies for hypertensive patients with obesity.

### The Combined Effects of Obesity and Hypertension on Left Ventricular Deformation Deterioration

The results of our study showed that hypertensive patients without obesity had significantly decreased strain values but preserved LVEF compared with control subjects, which confirmed the value of CMR-FT based myocardial deformation analysis in the detection of early and subtle LV dysfunction with previous studies (26, 27). A previous CMR-FT study (10) demonstrated decreased global longitudinal, circumferential and radial PS in essential hypertension. In the present study, the global strain decreased longitudinally but was preserved radially and circumferentially, consistent with an echocardiographic strain study by Sera (28). This difference suggests that longitudinal PS may be a more sensitive indicator than radial and circumferential PS for identifying the subclinical LV systolic dysfunction. This may be because the myocardium producing longitudinal stress mainly exists in the endocardium (29), which is generally reported to be the first site of myocardial ischemia (30).

Previous epidemiologic studies have provided substantial evidence that obesity could worsen cardiac function (31, 32). If there is hypertension at the same time, it also promotes the deterioration of cardiac function due to the chronic hemodynamic burden and central pressure overload (33). However, a previous study (34) showed that the presence of hypertension and obesity had no obvious effect on systolic function by LVEF. In this context, we have further examined the simultaneous presence of hypertension and obesity on the subclinical systolic function. Our results showed that in obese hypertensive patients, longitudinal PS was further decreased, and longitudinal PSSR began to be significantly impaired, despite comparable LVEF. These results suggest that subclinical systolic dysfunction caused by hypertension was further aggravated with obesity. This may be attributed to interrelated factors such as various neurohormonal and metabolic abnormalities, abnormalities in microvasculature and cardiac remodeling (4). In addition, these results also indicate that CMR-FT technique allows for early detection of subclinical systolic dysfunction, thus enabling timely intervention to prevent disease progression.

In addition, the occurrence of longitudinal and circumferential PDSR impairment in obese patients indicated an adverse effect of hypertension and combined obesity on subclinical diastolic dysfunction. Consistently, Kim et al. (34) demonstrated that obesity and hypertension intensified diastolic dysfunction by echocardiography. A previous study (35) showed that there was limited interaction on pathophysiological changes between obesity and hypertension in the development of diastolic function. Specifically, hypertension triggered apoptosis, inflammation and fibrosis, while obesity triggered oxidative stress and hypertrophic remodeling. The aggravation of subclinical LV diastolic dysfunction may provide a perspective in comorbidity-specific characterization.

### Associations Between Myocardial Perfusion, Left Ventricular Deformation and Body Mass Index in Hypertensive Patients

Multivariable stepwise regression analysis revealed that BMI exerted negative impacts on both myocardial perfusion and LV function. The negative effect of BMI on longitudinal PSSR and PDSR indicated that BMI was an important factor influencing LV systolic and diastolic function. In addition, we found that BMI was significantly associated with longitudinal PS in model adjustment with perfusion with improved model fit. With the increase in BMI, LV hypertrophic remodeling was noticeably intensified in hypertensive patients. Although the association between BMI and longitudinal PDSR lost significance after correction for myocardial perfusion, we assume that BMI is more likely to be higher in patients with lower perfusion, which also shows more severe diastolic dysfunction. Considering that microcirculation damage and subclinical LV systolic and diastolic dysfunction are predecessors of poor outcomes, these results emphasize the clinical implications of the adverse effects of obesity and the importance of weight management for hypertensive patients.

The present study found an independent association between TTM and longitudinal PSSR in hypertension, which is in accordance with previous evidence regarding diabetes by Liu et al. (6). Consistently, Li et al. demonstrated a significant association between impaired myocardial perfusion and subclinical systolic function in hypertension (24). In addition, previous studies have indicated an association between resting regional perfusion abnormalities and impaired diastolic function in consecutive patients who underwent single-photon emission computed tomography (SPECT) (36, 37). Our study also analyzed possible associations between perfusion and diastolic function in hypertension and found a similar association between upslope and longitudinal PDSR. These findings might suggest a possible mechanistic link between myocardial perfusion impairment by hypertension and subclinical LV systolic and diastolic dysfunction. Further investigation into underlying mechanisms and optimal treatment are warranted to improve LV function and prognosis for patients with essential hypertension.

### Limitations

There are several limitations of this study. First, this is a retrospective single-center study with inherent limitations. Therefore, further large-scale, multicenter, prospective research is needed to validate our results. Second, patient BMI was measured before CMR, and the predisease BMI and dynamic changes in BMI were not recorded and discussed in this study. Further studies are warranted to investigate the impact of dynamic changes in BMI on myocardial microcirculation and cardiac function. Third, the present study assessed only myocardial perfusion at rest, since stress perfusion has contraindications and potential risks. However, even in the resting state, the additive effect of hypertension and obesity on myocardial microcirculation has been proven.

## Conclusion

Obesity had an additive deleterious effect on myocardial microcirculation and LV function in patients with hypertension, and BMI was associated with myocardial microcirculation and LV function. Impaired myocardial perfusion was associated with subclinical LV dysfunction in hypertensive patients. These results emphasize the adverse effects of obesity and the importance of weight management for hypertensive patients and may imply mechanistic perspectives that could help in early diagnosis and the development of therapeutic strategies, thus improving prognosis for hypertensive patients.

## Data Availability Statement

The raw data supporting the conclusions of this article will be made available by the authors, without undue reservation.

## Ethics Statement

The studies involving human participants were reviewed and approved by Ethics Committee on Biomedical Research, West China Hospital of Sichuan University. The patients/participants provided their written informed consent to participate in this study.

## Author Contributions

P-LH: conceptualization, and writing—original draft. P-LH, X-ML, and W-FY: data curation. LJ, Y-KG, and YL: writing—review and editing. Z-GY and KL: supervision and writing—review and editing. Z-GY: funding acquisition. All authors contributed to the article and approved the submitted version.

## Conflict of Interest

The authors declare that the research was conducted in the absence of any commercial or financial relationships that could be construed as a potential conflict of interest.

## Publisher’s Note

All claims expressed in this article are solely those of the authors and do not necessarily represent those of their affiliated organizations, or those of the publisher, the editors and the reviewers. Any product that may be evaluated in this article, or claim that may be made by its manufacturer, is not guaranteed or endorsed by the publisher.
