# Supplementary material for: Additive Effects of Obesity on Myocardial Microcirculation and Left Ventricular Deformation in Essential Hypertension: A Contrast-Enhanced Cardiac Magnetic Resonance Imaging Study
Source: Front Cardiovasc Med. 2022 Mar 24;9:831231. doi: 10.3389/fcvm.2022.831231 (PMC8987987; doi:10.3389/fcvm.2022.831231)
Supplement: Supplementary file 1 [file Data_Sheet_1.pdf]

Additive effects of obesity on myocardial microcirculation and left ventricular deformation in essential hypertension: a contrast-enhanced cardiac magnetic resonance imaging study

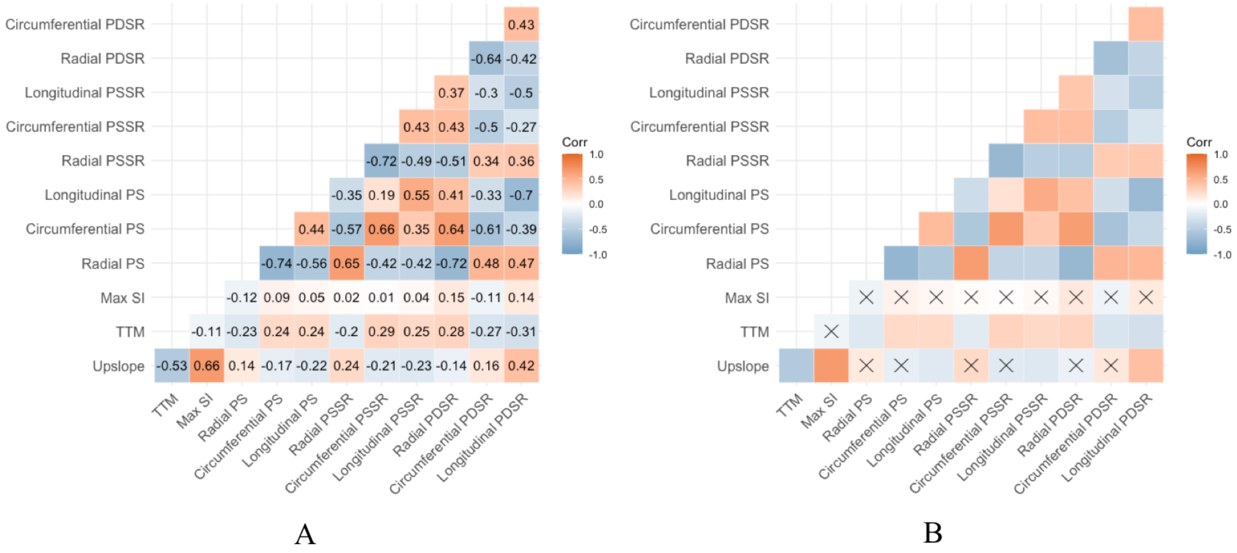

Supplementary Figure 1. Correlations between myocardial perfusion parameters and LV strains in hypertensive patients. Colors represents the correlation coefficients and its intensity represents the coefficient's value (A) and × indicates correlation is non-significant (B). Abbreviations are as in table 2.

Supplementary Table 1. Univariable linear regression analysis in hypertensive patients

	Upslope		TTM		Max SI		Longitudinal PS		Longitudinal PSSR		Longitudinal PDSR	
	**β (95% CI)**	**P**	**β (95% CI)**	**P**	**β (95% CI)**	**P**	**β (95% CI)**	**P**	**β (95% CI)**	**P**	**β (95% CI)**	**P**
**Age**	-0.004 (-0.015, 0.008)	0.536	0.105 (-0.066, 0.276)	0.226	0.065 (-0.035, 0.165)	0.201	-0.006 (-0.046, 0.034)	0.765	0.001 (-0.001,0.004)	0.328	0.004 (-0.002,0.010)	0.152
**Sex**	-0.176 (-0.531, 0.180)	0.329	4.673 (-0.499,9.85)	0.076*	0.205 (-2.869, 3.279)	0.895	2.214 (1.086, 3.341)	<0.001*	0.097 (0.018, 0.175)	0.016*	-0.150 (-0.237, -0.063)	0.001*
**BMI**	-0.136 (-0.189, -0.084)	<0.001*	0.392 (-0.471, 1.254)	0.370	-0.903 (-1.376, -0.430)	<0.001*	0.372 (0.187, 0.557)	<0.001*	0.019 (0.007, 0.032)	0.003*	-0.030 (-0.044, -0.017)	<0.001*
**Heart rate**	0.002 (-0.009, 0.014)	0.670	-0.141 (-0.306, 0.024)	0.092*	-0.086 (-0.182, 0.011)	0.080	0.011 (-0.028, 0.049)	0.576	-0.002 (-0.004, 0.001)	0.199	-0.001 (-0.004, 0.002)	0.359
**Office SBP**	-0.001 (-0.010, 0.007)	0.796	-0.047 (-0.172, 0.078)	0.454	-0.008 (-0.081, 0.065)	0.830	0.033 0.005, 0.061)	0.022*	-0.000 (-0.002, 0.002)	0.979	-0.002 (-0.004,0.000)	0.085*
**Office DBP**	-0.003 (-0.014, 0.008)	0.552	-0.095 (-0.252, 0.062)	0.235	-0.059 (-0.150, 0.033)	0.209	0.026 (-0.010, 0.062)	0.152	-0.001 (-0.003, 0.002)	0.655	-0.002 (-0.005, 0.001)	0.178

**TG**	-0.056 (-0.165, 0.052)	0.305	0.859 (-0.770, 2.49)	0.298	-0.264 (-1.210, 0.682)	0.581	0.330 (-0.034, 0.693)	0.075*	0.018 (-0.007, 0.043)	0.153	-0.023 (-0.051, 0.005)	0.111
**HDL**	0.017 (-0.146, 0.180)	0.838	0.440 (-2.005, 2.885)	0.722	0.230 (-1.185, 1.644)	0.748	-0.090 (-0.642, 0.462)	0.747	-0.06 (-0.092, -0.021)	0.002*	0.024 (-0.018, 0.066)	0.259
**eGFR**	0.000 (-0.008, 0.008)	0.973	0.067 (-0.062, 0.196)	0.305	-0.019 (-0.079, 0.041)	0.529	0.000 (-0.029, 0.030)	0.960	0.000 (-0.002, 0.002)	0.808	0.000 (-0.002, 0.002)	0.854
**CCB**	-0.139 (-0.496, 0.218)	0.442	0.168 (-5.100, 5.437)	0.950	-1.733 (-4.795, 1.329)	0.264	0.946 (-0.253, 2.145)	0.120	-0.003 (-0.083, 0.078)	0.945	-0.050 (-0.142, 0.041)	0.277
**LVEF**	-0.010 (-0.037, 0.018)	0.488	0.025 (-0.380, 0.429)	0.903	-0.240 (-0.471, -0.008)	0.043*	-0.175 (-0.261, -0.089)	<0.001*	-0.007 (-0.013, 0.001)	0.023*	0.009 (0.002, 0.016)	0.013*
**LVEDVI**	0.003 (-0.006, 0.012)	0.480	0.012 (-0.119, 0.143)	0.853	0.042 (-0.035, 0.118)	0.283	0.042 (0.013, 0.071)	0.005*	0.003 (0.001, 0.005)	0.005*	-0.001 (-0.003, 0.001)	0.452
**LVESVI**	0.012 (-0.04, 0.029)	0.127	-0.056 (-0.297, 0.185)	0.646	0.125 (-0.014, 0.264)	0.077*	0.067 (0.013, 0.121)	0.016*	0.004 (0.000, 0.007)	0.042*	-0.002 (-0.006, 0.003)	0.469

**LVMI**	0.000 (-0.008, 0.009)	0.913	-0.023 (-0.149, 0.100)	0.696	0.031 (-0.042，0.104）	0.398	0.062 (0.036, 0.088)	<0.001*	0.002 (0.000, 0.004)	0.027*	-0.003 (-0.005, -0.001)	0.015*
**LV remodeling index**	-0.303 (-1.103, 0.498)	0.455	-2.252 (-14.062, 9.558)	0.706	0.020 (-6.892, 6.932)	0.995	3.839 (1.226, 6.452)	0.004*	0.030 (-0.151, 0.211)	0.743	-0.284 (-0.483, -0.086)	0.005*
**Upslope**	-	-	-	-	-	-	-0.737 (-1.398, -0.077)	0.029*	-0.051 (-0.095, -0.007)	0.023*	0.109 (0.063, 0.156)	<0.001*
**TTM**	-	-	-	-	-	-	0.056 (0.011, 0.100)	0.015*	0.003 (0.001, 0.007)	0.011*	-0.005 (-0.009, -0.002)	0.002*
**Max SI**	-	-	-	-	-	-	0.021 (-0.058, 0.099)	0.601	0.001 (-0.004, 0.006)	0.675	0.004 (-0.002, 0.010)	0.152

Abbreviations are as in table 1 and table 2.

*P<0.1
